# Human proximity seeking in family pigs and dogs

**DOI:** 10.1038/s41598-020-77643-5

**Published:** 2020-11-30

**Authors:** Paula Pérez Fraga, Linda Gerencsér, Attila Andics

**Affiliations:** 1 Department of Ethology, Eötvös Loránd University (ELTE), Pázmány P. s. 1/C, 1117 Budapest, Hungary; 2 MTA-ELTE ‘Lendület’ Neuroethology of Communication Research Group, Eötvös Loránd University (ELTE), Budapest, Hungary

**Keywords:** Evolution, Zoology

## Abstract

Family dogs (*Canis familiaris*) seek human contact from an early age, can discriminate and prefer their caregivers over other humans. To investigate if being kept as a family animal is sufficient to induce similar early human proximity-seeking in another domestic mammal, here we directly compared such behaviours in dogs and similarly raised domestic pigs (*Sus scrofa domesticus*). We used a preference test to measure proximity-seeking behaviours of 4-month-old family pigs and dogs in the presence of their caregiver and either a stranger or a familiar object, in a novel environment. We found that both pigs and dogs preferred their caregivers over the familiar object but not over the stranger. However, when the caregiver and the stranger were present, only dogs showed an overall preference for human proximity, and pigs spent more time away from both humans. These results suggest that both dogs and pigs seek the proximity of their caregiver, but there is a difference in how each species generalizes their experience to other humans. Species-specific predispositions, including that dogs have a longer socialization period and that humans are more salient as a social stimulus for them, may be important for the early development of an overall preference for humans.

## Introduction

Domestic animals live close to and depend on humans, typically they show increased tolerance to human proximity, and are less reactive to human intervention compared to their wild relatives^1–4^ . Behavioural reactions of both companion and farm animals to human presence have been widely investigated^5–7^, with a special focus on approach and maintaining proximity. Approaching humans can have different functions, such as exploration, grouping, displaying threat or can be controlled by specific incentives, such as food or social contact^8,9^. In most contexts this approach behaviour and subsequent proximity is associated with a lower level of fear^6,7,10^, consequently more fearful animals are less likely to approach humans (review:^11^).

Dogs (*Canis familiaris*) show a tendency of approaching and interacting with humans defined as sociability^12,13^, which is one of the most important factors in dog–human relationship^5^. It has been assumed that the propensity to approach humans in the absence of fear could have been selected for during domestication^5,14,15^. This predisposition is reflected in the fact that dogs with limited exposure to human presence are reported to seek human proximity^16–18^. However, socialisation to humans during the sensitive phase has an important role in the development of human preference^19^. Furthermore, it is widely accepted that family dogs discriminate their owners from familiar and unfamiliar humans^20,21^ and that they form attachment bonds with their owner from an early age^22,23^.

Dogs’ approach behaviour to humans was observed in a preference test, in which intensively socialized 4-week-old puppies preferred to stay in proximity of their caregiver versus an unfamiliar person or a conspecific^14^. However, after one week, young family dogs only showed preference for the caregiver when the other choice was a conspecific and displayed a general human preference when the caregiver was present with an unfamiliar human (i.e. spending more time in proximity of both familiar and unfamiliar humans). In the same study, this behavioural change was not displayed by similarly raised wolves. Differently to dog puppies, 5-week-old wolves showed no preference towards their caregiver when the other choice was an adult dog, but they preferred the caregiver when she was present together with an unfamiliar human. This difference might be based on how both species recognize a familiar stimulus and how they generalize to other stimuli^14,24^. A similar outcome was observed in another study, in which adult family dogs and similarly raised wolves were exposed to a familiar person and a stranger^5^. Individuals of both species stayed longer in proximity of the familiar human, but dogs showed a higher preference to interact and stay in close proximity with both familiar and unfamiliar humans. The general preference for close proximity to humans seems to be a species-specific predisposition in dogs that develops as a result of early socialisation. This tendency for social interactions is the foundation for development of attachment to humans^16^ that does not emerge in young wolves^23^.

Although approaching humans has been investigated in many domestic species^7,10,25^, no direct comparisons have been made with family dogs. As experience with humans seems to be an important factor for the development of approach behaviour^19,26,27^, testing another highly social domestic animal, while keeping the rearing conditions similar, could add valuable information about the contribution of species-specific predispositions and environmental factors.

The popularity of the domestic pig (*Sus scrofa domesticus*) as a companion animal, especially that of the miniature variant, has grown during the past years^28^. We should take into special consideration this relatively new role of the domestic pig, which creates the need to better understand the pig-human relationship in a household environment. As companion animals, pigs are social, accommodate relatively well to the family environment and can be trained^29^. Along with this, the domestic pig seems to be a good candidate for direct comparison with the dog as it is also a social group-living species, and its domestication history^30^ shows many parallels with the emergence of dogs^15^. Unlike other farm animals, pigs are omnivorous, which might have attracted their ancestors to the leftover food around human settlements, and similarly to dogs’ ancestors, their presence was tolerated as a livestock animal and for recycling waste^31,32^. Interestingly, besides the meat stock purpose, in some cultures pigs have been used until nowadays for rooting in the gardens, and occasionally young pigs have been treated as companion animals^33,34^. Nevertheless, interest in pigs’ behavioural reactions towards humans has been focused on pigs kept under farm/laboratory conditions, and scarce research has been done on pigs kept as companion animals^35,36^.

Behaviour data from observations under farm conditions show that young domestic pigs approach and try to make contact with humans readily as part of exploring the environment or to make social contact^37,38^. Positive handling, that is gently stroking the pig’s head and body, increased pigs’ propensity to approach and interact with the human handler, reflecting decreased fear levels^8,39^. Furthermore, pigs are not only able to recognize familiar over unfamiliar conspecifics, which allows them to form and maintain stable social groups^40,41^, but they also discriminate familiar humans from unfamiliar ones^42–44^. However, no data is available about the behaviour of pigs living in human families toward their caregiver.

Here we provided young miniature pigs with a similar intensive human socialization as family dogs, which allowed us to use a comparative framework and directly contrast the two species’ approach behaviour to humans. Our aim was to investigate the role of species-specific predispositions in the two species’ reactions towards the presence of their caregiver. For that, we presented the subjects with a preference test, in which the owner was either paired with an unfamiliar person or with a familiar object. We measured the approach and proximity seeking of both piglets and dog puppies. We hypothesized that family pigs would exhibit preference for their caregiver similarly as family dogs do. Furthermore, we assumed that family dogs would display more proximity seeking behaviours towards an unfamiliar human than family pigs.

## Materials and methods

### Ethical statement

We received official approval (# PE/EA/430-6/2018) for the experimental protocols from the local ethical committees: Állatkísérleti Tudományos Etikai Tanács (Scientific Ethic Council of Animal Experiments); Pest Megyei Kormányhivatal Élelmiszerlánc-Biztonsági és Állategészségügyi Igazgatósága, Budapest, Hungary (Food Chain Safety and Animal Health Directorate Government Office). We also received the necessary permission from the University Institutional Animal Care and Use Committee (UIACUC, Eötvös Loránd University, Hungary).

### Subjects

We recruited volunteer pig owners for participation, and all the pig subjects were exposed to close human contact in families from ~ 8 weeks of age. Since the pigs we used in this study are enrolled in a long-term scientific project (http://etologia.elte.hu/en/lendulet-neuroethology-of-communication/), they were the same subjects as in a previously published work, so a more detailed description about their rearing conditions can be found there^35^. The dogs’ owners were volunteers of the Family Dog Project (https://familydogproject.elte.hu/). We asked the owners about the dogs’ rearing conditions prior to enrolment to make sure that their socialization background was similar to that of the pigs. Owners provided a written consent form to voluntary permit their dogs and pigs to participate in the study, as well as to publish their images and data.

Our subjects were ~ 4 months old juvenile family pigs (*N* = 9; 6 neutered males and 3 intact females; X_age_ ± SD = 3.8 ± 0.9 months; Minnesota and Minnesota-mixed miniature variants) and dogs (*N* = 12; 5 intact males and 7 females; X_age_ ± SD = 3.7 ± 0.7 months; from 8 different breeds, including poodles and collies) (see Supplementary material Table S1, for detailed information about the subjects).

### Procedure

We carried out this study in a test room (4.45 m × 3.68 m room) of the Department of Ethology (Eötvös Loránd University, Hungary) in the presence of the animals’ owners. No signs of excessive distress or fear, were shown by any of the subjects during this study, similar as in our previous work^35,36^.

We partly based our method on the work done by Gácsi and colleagues (2005). The subjects were presented with object-preference tests in two consecutive conditions, the order of which was balanced across the subjects. The two ‘targets’ in one condition were the caregiver (C) simultaneously presented with either a stranger (S) (i.e. a female experimenter unknown to the subjects) or a familiar object (O), which was their own, clean and empty feeding bowl. The test began with, and the two conditions were also separated by a 5-min *isolation period*. During this time the subject was completely isolated from social contact in its own carrier box outside of the test room. This was a prerequisite to elicit similar motivation to make social contact in both species.

### Caregiver–Stranger condition (C–S) (5 min)

**Figure 1:**
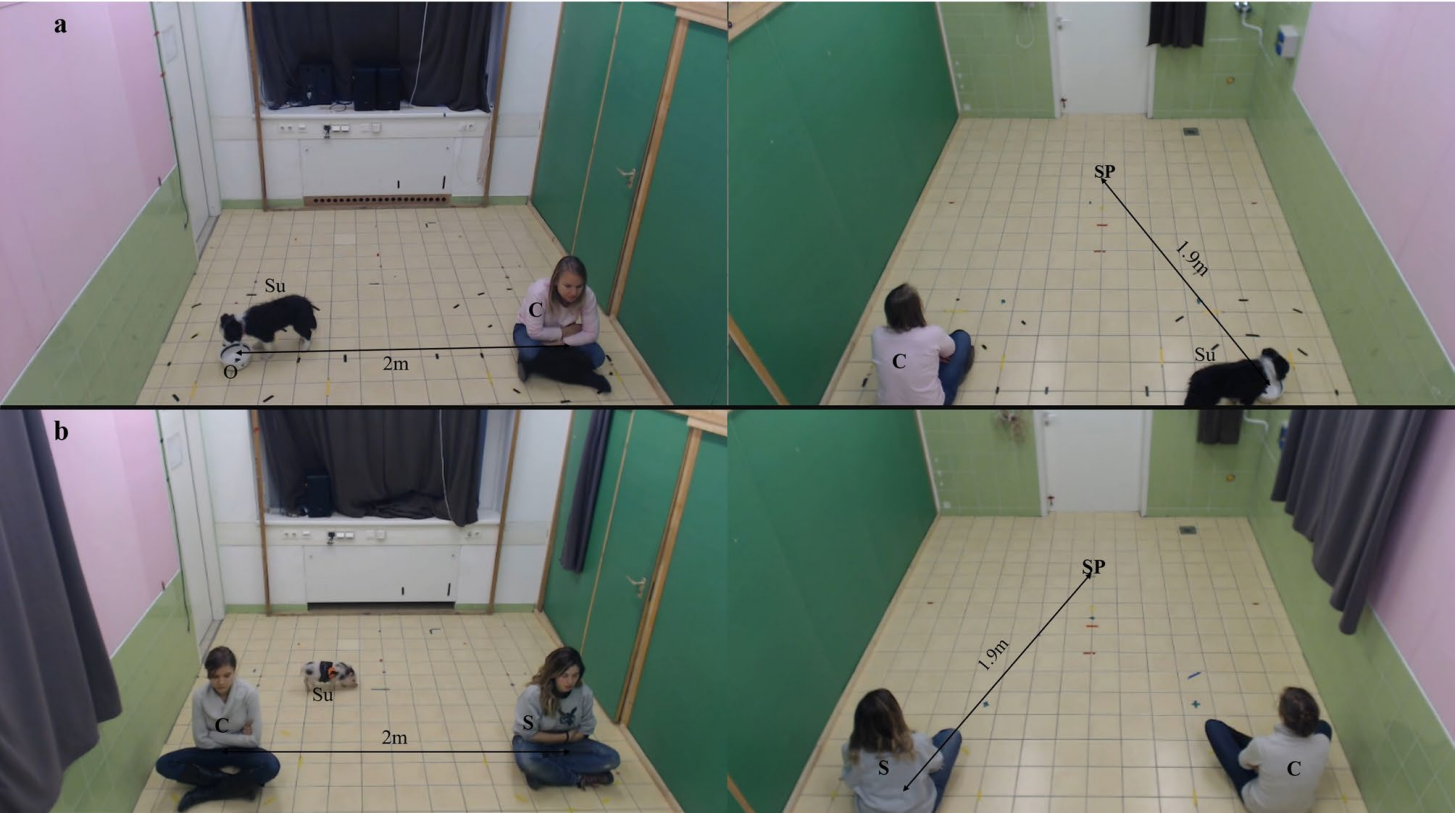
Experimental setup of the preference test, modified from Gácsi et al. (2005). *Su* subject, *C* caregiver, *S* stranger, *O* object, *SP* subject’s starting point. (**a**) Caregiver–Object condition, (**b**) Caregiver–Stranger condition. Informed consent to publish was obtained from these owners/participants.

Apart from subject, C and S were present in the otherwise empty room. The two humans were both passively and quietly sitting cross-legged on the floor with their arms folded in front of their chest. Their positions were predetermined; one of two possible locations 2 m apart from each other, forming together an equilateral triangle with and facing towards the starting position of the subject, that was at a distance of 1.9 m from both of them (see Fig. 1). At the beginning of the test, another experimenter carried the subject into the room, positioned it at its starting point facing the humans, let it free and then left the room. The behaviour of subject was not restricted in any way throughout the 5 min duration of the test.

### Caregiver–Object condition (C–O) (5 min)

Subject, C and O were present in the otherwise empty room. The whole procedure—apart from the presence of O instead of S—followed the above description in the Caregiver–Stranger condition (see Fig. 1). The positions of C, S and O were balanced between the two conditions and counterbalanced across subjects.

### Behavioural analysis

All tests were video-recorded for later behavioural analysis using Solomon Coder (v. 090,913; András Péter http://solomoncoder.com). We measured the following behaviours during the 300 s measured from the moment the subject was let free at the starting point:

*Near caregiver* (duration, s): Any body part of the subject is within a distance of 40 cm from C with or without establishing physical contact with the human;

*Contact caregiver* (duration, s): The subject is establishing physical contact with C;

*Near stranger/object* (duration, s): Any body part of the subject is within a distance of 40 cm from either S (Caregiver–Stranger condition) or O (Caregiver–Object condition) with or without establishing physical contact with the human/object;

*Contact stranger/object* (duration, s): The subject is establishing physical contact with S or O;

*Contact ratio for caregiver* (value between 0 and 1)*:* The ratio of time spent in physical contact with C out of the total time spent near C (within a distance of 40 cm). We used the following formula: ‘*Contact caregiver*’*/*‘*Near caregiver*’;

*Contact ratio for stranger/object* (value between 0 and 1): The ratio of time spent in physical contact with S or O out of the total time spent near S or O (within a distance of 40 cm), respectively. We used the following formula: ‘*Contact stranger/object*’*/*‘*Near stranger/object*’;

*Away* (duration, s): The subject is at least 40 cm distance away from C or S/O;

*Latency to first approach the *‘*targets*’ (s): The time taken from the moment that the subject is let free till it first approaches either of the targets (C, S or O) within a distance of 40 cm;

*Preference index for caregiver* (value between − 1 and + 1): for the calculation we considered the total time spent in proximity of the caregiver (with and without physical contact). Following the method of Gácsi and colleagues^14^ it was calculated as follows: [(‘Near caregiver (total)’ – ‘Near stranger/object (total)’)/(‘Near caregiver (total)’ + ‘Near stranger/object (total)’)]. A value close to zero indicates no preference (in terms of total time spent in proximity), while a value closer to + 1 or − 1 indicates high preference for the caregiver or stranger/object, respectively;

*Preference index for social proximity* (value between − 1 and + 1): based on the same method as described above, this index was calculated as follows: [(total time spent near social partners – total time spent away from social partners)/(total time spent near social partners + total time spent away from social partners)]. The social partners were either C alone (Caregiver–Object condition) or both C and S (Caregiver–Stranger condition);

*Return to caregiver* (frequency count): the number of times the subject approaches C closer than 40 cm (the obvious pre-requisite of approaching more than once is going further away after the previous approach).

*Return to stranger/object* (frequency count): the number of times subject approaches S (Caregiver–Stranger condition) or O (Caregiver–Object condition) within 40 cm;

*Vocalization* (duration, s): subject is emitting any vocalizations (e.g., barks, whines for dogs; grunts for pigs);

*Vocalization near social partner* (duration, s): concurrence of vocalization and being near C and/or S (both with or without physical contact).

The recordings were coded manually by one main coder for the variables ‘Near caregiver’, ‘Contact caregiver’, ‘Near stranger/object’, ‘Contact stranger/object’ and ‘Away’. The frequency values for the variables ‘Return to caregiver’ and ‘Return to stranger/object’ were automatically derived by the coding software (from the uninterrupted occurrences of ‘Near caregiver and stranger/object’). Twenty percent of the recordings was also coded by a secondary coder for the manually coded variables. We calculated the agreement between the two raters based on the raw coding sheets, where the variables were coupled together into two variables with more levels, i.e. ‘Near caregiver’, ‘Near stranger/object’ and ‘Away’ into Position, and ‘Contact caregiver’ and ‘Contact stranger/object’ into Contact. The interrater agreement was near perfect for both Position (Cohen’s Kappa, κ = 0.96, *P* < 0.001 for pigs and κ = 0.95, *P* < 0.001 for dogs) and Contact (Cohen’s Kappa, κ = 0.87, *P* < 0.001 for pigs and κ = 0.89, *P* < 0.001 for dogs). The vocalization durations could be determined without ambiguity based on the sonograms belonging to the video recordings, thus we did not calculate interrater agreement for these variables.

### Data analysis

Data analysis was performed using R statistical environment (v. 3.5.0. R Development Core Team). We tested for the main effects of Species (between-subject factor), Condition (within-subject factor) and the interaction of these two factors on the following dependent variables: ‘Near caregiver and stranger/object’, ‘Contact caregiver and stranger/object’, ‘Contact ratio for caregiver and stranger/object’, ‘Away’ and ‘Return to caregiver and stranger/object’. We tested for the main effects of ‘Species’, ‘Condition’ and ‘Target’ (C, S and O, within-subject factor) and the interaction of these three factors on the variable ‘Latency to first approach the targets’.

We only reported main effects in absence of interaction of any of the factors. For the continuous variables that did not follow normal distribution (i.e. ‘Near caregiver and stranger/object’, ‘Contact caregiver and stranger/object’ as indicated by Shapiro–Wilk tests) we applied the method of Box-Cox power transformations with optimal lambda parameters to fulfil normality criteria. For normally distributed data we built Linear Mixed-effects Models (LMMs) fit by residual maximum likelihood (REML) and used Satterthwaite approximation for estimating the degrees of freedom, while for Poisson-distributed count data we built Generalized Linear Mixed-effects Models (GLMMs) fit by maximum likelihood using Laplace Approximation. We built Mixed Effect Cox Regression models (coxme function) for the ‘Latency to first approach the targets’. We included individual subjects as a random factor in all the models and obtained pairwise post-hoc comparisons for the fixed factors, which we reported only in the presence of interaction of the fixed factors. We report the results of the final models. To test for divergences from zero of the preference indices we used either One sample *t* tests (for normally distributed data) or One sample Wilcoxon signed-rank tests (for non-normally distributed data).

We performed quantitative analysis on the vocalization data. Half of the dog subjects did not produce any vocalizations in either conditions and two of them vocalized for less than 1 s duration in one condition, so we further analysed the vocalizations of the pigs only. To test for differences between the relative duration of vocalization near and away from the social partner(s) in both conditions (i.e. duration of vocalization near social partner(s)/duration of being near social partner(s) with or without physical contact) we used Wilcoxon signed-rank tests.

The datasets generated and analysed during the current study are available in the form of electronic supplementary material (Supplementary Dataset).

### Ethical approval

All applicable international, national, and institutional guidelines for the care and use of animals were followed. All procedures performed in both animal studies were in accordance with the ethical standards of the institution at which the studies were conducted.

## Results

Detailed results of the GLMMs and the results of post-hoc comparisons are shown in Supplementary material (Tables S2–S6).

### Time spent in proximity and away from the social partners/object

**Figure 2:**
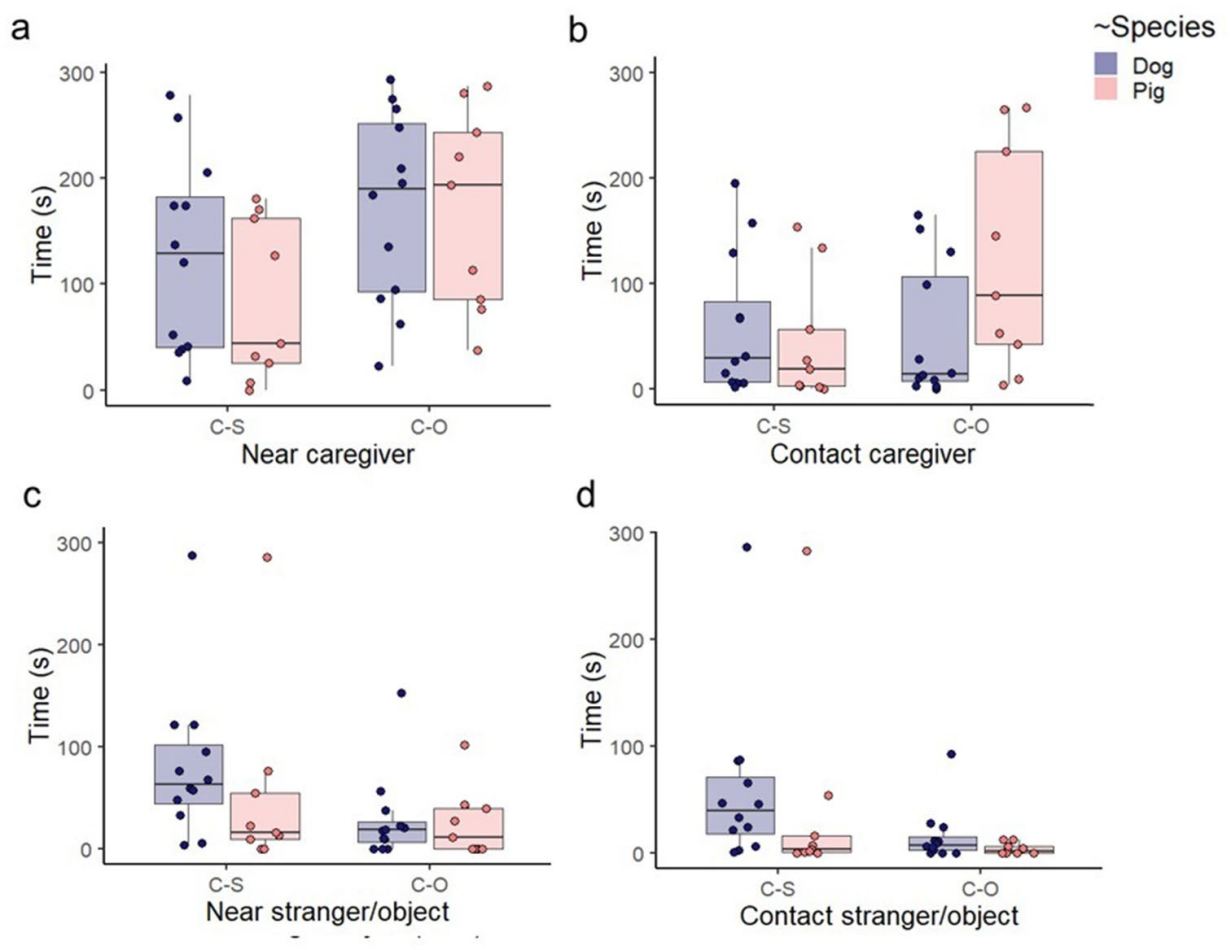
Time spent near the caregiver [(**a**) in total, (**b**) in contact] and the stranger/object [(**c**) in total, (**d**) in contact] for both species in the two conditions. “C–S” stands for Caregiver–Stranger condition and “C–O” for Caregiver–Object condition. The line across the box represents the sample median, the box represents the interquartile range, and the whiskers show the smallest and largest values (excluding outliers). The dots represent the individual data points. See also Supplementary material Table S2.

Both species spent more time near the caregiver (with and without physical contact) in the Caregiver–Object condition than in the Caregiver–Stranger condition (LMM, main effect of ‘Condition’: *F*_1,19_ = 12.673, *P* = 0.002). (Fig. 2a). There was a significant interaction between Species and Condition (Caregiver–Stranger vs Caregiver–Object) on the time spent with ‘Contact caregiver’ (LMM, *F*_1,19_ = 5.48, *P* = 0.03). Pigs in the Caregiver–Object condition tended to spend more time in physical contact with the caregiver than in the Caregiver–Stranger condition (post hoc comparison, *P* = 0.084, Supplementary material Table S2) (Fig. 2b). The interaction between Species and Condition on the ‘Contact ratio for caregiver’ was also significant (LMM, *F*_1,19_ = 8.14, *P* = 0.01). In the Caregiver–Object condition, the ratio of time spent in physical contact with the caregiver tended to be higher for pigs than for dogs (post hoc comparison, *P* = 0.081, Supplementary material Table S3) (Fig. S1a).

Condition had a main effect on ‘Near stranger/object’ (LMM, *F*_1,19_ = 5.325, *P* = 0.027), meaning that both species spent more time (with and without physical contact) in the proximity of the stranger than the familiar object (Fig. 2c). Dogs spent more time than pigs in physical contact with both the stranger and the familiar object (LMM, main effect of Species, *F*_1,19_ = 6.319, *P* = 0.016), while both pigs and dogs spent more time contacting the stranger than the familiar object (LMM, main effect of Condition, *F*_1,19_ = 5.507, *P* = 0.024) (Fig. 2d). There was a main effect of both Species (LMM, *F*_1,19_ = 9.27, *P* = 0.004) and Condition (LMM, *F*_1,19_ = 6.39, *P* = 0.02) on the ‘Contact ratio for stranger/object’; meaning that from the total time spent near the stranger/object dogs spent more time than pigs in physical contact with both the stranger and the object, while both species spent more time in physical contact with the stranger than the object (Fig. S1b).

**Figure 3:**
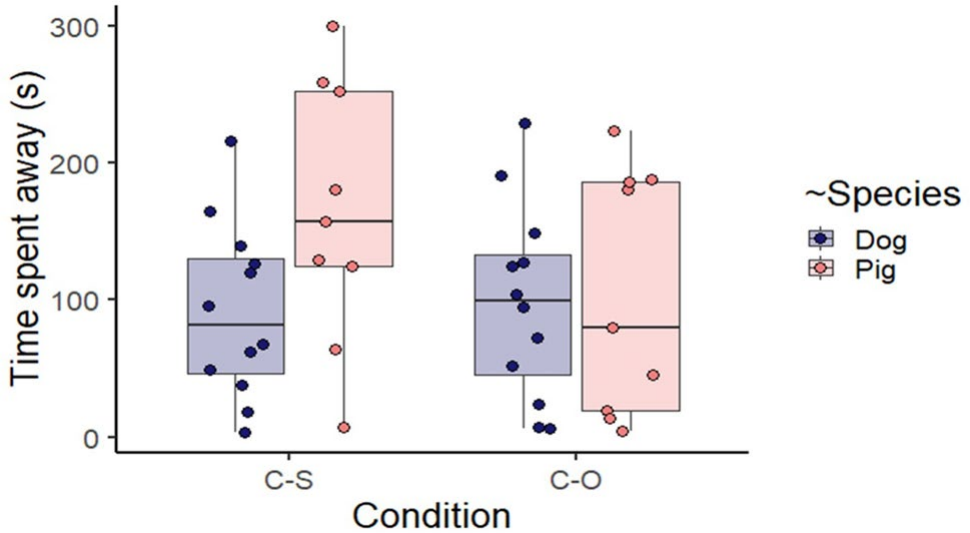
Time spent away from the persons/object for both species in the two conditions. “C–S” stands for Caregiver–Stranger and “C–O” for Caregiver–Object. The line across the box represents the sample median, the box represents the interquartile range, and the whiskers show the smallest and largest values (excluding outliers). The dots represent the individual data points. See also Supplementary material Table S4.

The interaction between ‘Species’ and ‘Condition’ affected significantly the time spent ‘Away’ (LMM, *F*_1,19_ = 7.193, *P* = 0.015); pigs but not dogs, spent more time away from the stimuli in the Caregiver–Stranger condition than in the Caregiver–Object condition (post hoc comparison, *P* = 0.023, see Supplementary material Table S4) (Fig. 3).

### Latency to first approach the ‘targets’

‘Species’ had a main effect (Cox, β ± SE = − 1.49 ± 0.31, p < 0.001), proving that pigs approached any of the targets in both conditions later than dogs. The interaction between ‘Target’ and ‘Condition’ proved to affect the ‘Latency to first approach the targets’ significantly (X^2^_2_ = 11.22, p < 0.001); both pigs and dogs approached the caregiver earlier than the familiar object (post-hoc comparison for caregiver vs. familiar object: β ± SE = − 1.97 ± 0.39, p < 0.001), but not earlier than they approached the stranger (post-hoc comparison for caregiver vs. stranger: β ± SE = − 0.21 ± 0.33, *p* = 0.52).

### Preference indices for the caregiver and for social proximity

**Figure 4:**
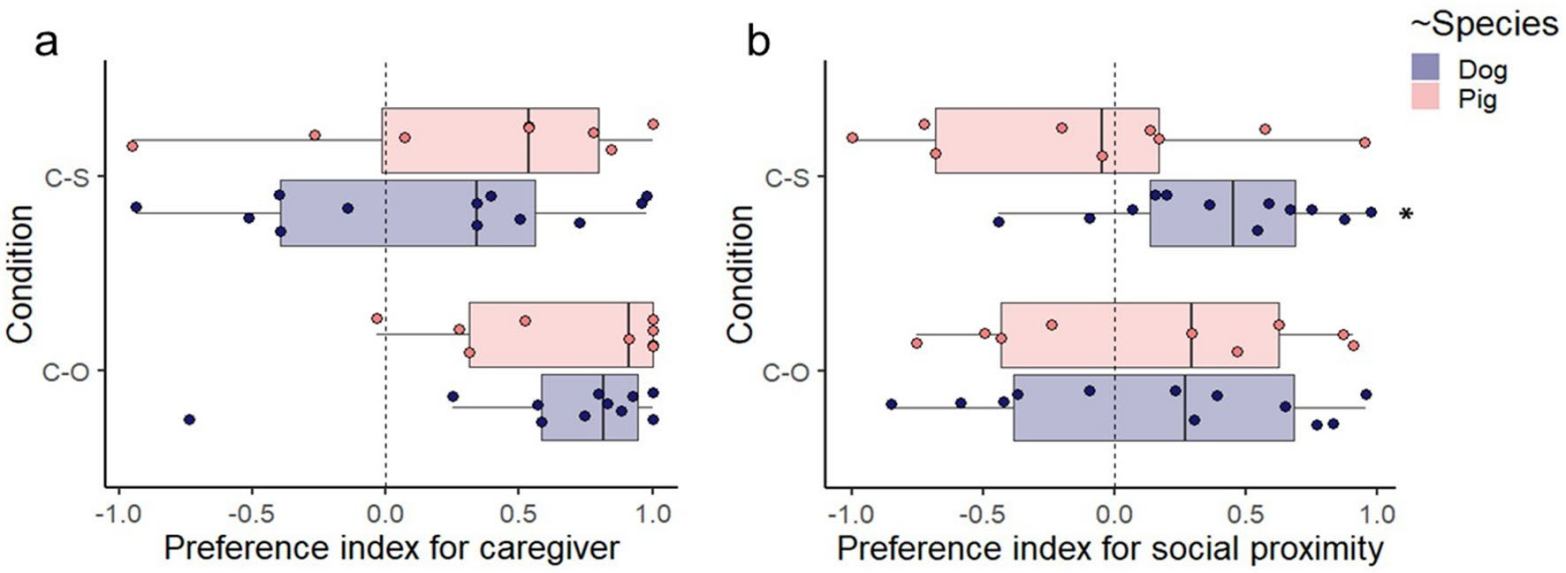
Pigs’ and dogs’ preference indices for the caregiver (**a**) and for social proximity (**b**) in the two conditions. The line across the box represents the sample median, the box represents the interquartile range, and the whiskers show the smallest and largest values (excluding outliers). “C–S” stands for Caregiver–Stranger and “C–O” for Caregiver–Object.

Neither pigs’ nor dogs’ ‘Preference index for the caregiver’ differed significantly from zero in the Caregiver–Stranger condition (One sample Wilcoxon signed-rank tests*, W* = 48, df = 11, *P* = 0.519 for dogs and *W* = 27, df = 8, *P* = 0.25 for pigs), whereas these indices showed a significant preference for the caregiver in the Caregiver–Object condition in both pigs and dogs (One sample Wilcoxon signed-rank tests, *W* = 74, *P* = 0.007 for dogs and *W* = 44, *P* = 0.012 for pigs) (Fig. 4a). The ‘Preference index for social proximity’ showed significant difference from zero for dogs but not for pigs in the Caregiver–Stranger condition (One sample *t* tests, *t* = 3.18, df = 11, *P* = 0.009 for dogs and *t* = -0.432, df = 8, *P* = 0.677 for pigs), and it did not differ from zero in the Caregiver–Object condition for either species (One sample *t* tests, *t* = 0.87, *P* = 0.403 for dogs, *t* = 0.663, *P* = 0.525 for pigs) (Fig. 4b).

### Return to caregiver and to stranger/object

**Figure 5:**
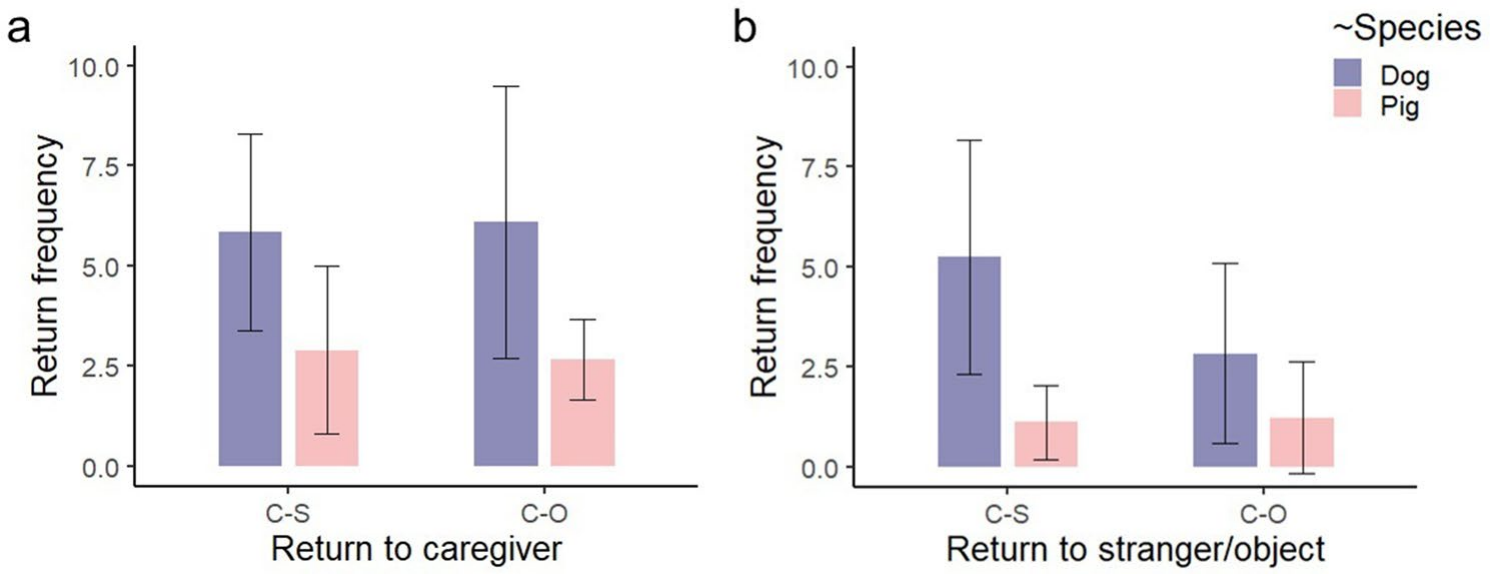
Pigs’ and dogs’ return frequencies to the caregiver (**a**) and to the stranger/object (**b**) during the 5 min test in the two conditions. The boxes represent the mean values and the whiskers show the corresponding standard deviations. “C–S” stands for Caregiver–Stranger condition and “C–O” for Caregiver–Object condition. See also Supplementary material Table S5.

There was significant main effect of Species on the frequency of ‘Return to caregiver’ (GLMM, *Z* = − 3.026, *P* = 0.003, see also Supplementary material Table S5); meaning that pigs returned to the caregiver less frequently than dogs in both conditions (see Fig. 5a). Both Species (GLMM, *Z* = − 2.066, *P* = 0.039) and Condition (GLMM, *Z* = 2.898, *P* = 0.004) had a significant main effect on the frequency of ‘Return to stranger/object’ (see also Supplementary material Table S6); pigs returned less frequently to the stranger/object than dogs in both conditions and the frequency of return to the stranger was higher than the frequency of return to the object for both dogs and pigs (see Fig. 5b).

### Vocalizations

Only 4/12 dogs vocalized during the C–O condition and 6/12 during the C–S condition. All pigs vocalized in both conditions, so we further analyse only the vocal behaviour of the pigs. Pigs showed weak tendencies to vocalize less near (X_C–O_ ± SD = 0.06 ± 0.07; X_C–S_ ± SD = 0.08 ± 0.09) than away (X_C–O_ ± SD = 0.26 ± 0.39; X_C–S_ ± SD = 0.15 ± 0.12) from the social partner(s) in both conditions (Wilcoxon signed-rank tests, *W* = 31, df = 8, *P* = 0.078 and *W* = 37, df = 8, *P* = 0.098 in Caregiver–Stranger and Caregiver–Object conditions respectively).

## Discussion

To the authors’ present knowledge this is the first report on young miniature family pigs’ spontaneous reactions to the presence of their caregiver paired with a stranger or a familiar object, compared directly to the behaviour of family dogs. Both family pigs and dogs seek for the proximity of their caregiver in unrestricted neutral contexts, as reflected by the similar total time spent and preference for being in direct vicinity of them. Both species preferred to stay in proximity of the caregiver versus the familiar object, but neither pigs nor dogs preferred their caregiver versus the stranger. Along with this, both species approached earlier the caregiver than the familiar object, but not earlier than the stranger. Nevertheless, despite these similar responses to the presence of their caregiver, several differences have arisen between the two species’ proximity seeking behaviours which seem to be more context-dependent for pigs than for dogs. First of all, pigs spent more time in physical contact with the caregiver during the Caregiver–Object condition and in this condition they tended to do so more than dogs. Interestingly, pigs—but not dogs—spent more time away from the two stimuli (caregiver and stranger or object, respectively) in the Caregiver–Stranger condition than in the Caregiver–Object condition. This difference is also reflected in the finding that dogs, but not pigs preferred to stay in social proximity (i.e. being near a social partner other than anywhere else in the room) when the caregiver and the stranger were present. Additionally, we found that pigs returned less frequently both to the caregiver and the stranger than dogs. Furthermore, all pigs, but not all dogs, vocalized in both conditions and they showed a weak tendency to vocalize less while being near than away from a social partner.

The similar amount of time near the caregiver found in dogs and miniature pigs is in line with our hypothesis that intensively socialized young family pigs would show analogous behaviour as young family dogs in the presence of their caregiver. Although farm pigs are motivated to approach humans^11^, our results are the first to show that young family pigs seek the proximity of their caregiver in a novel environment. Different factors can explain why piglets approach and stay in the proximity of their caregiver. Familiarity and positive previous experience with the owner may encourage animals to approach him/her^11^ as his/her presence could predict positive events^45,46^. It may be advantageous for young animals to choose a familiar stimulus over an unfamiliar one when exposed to an unfamiliar situation^47,48^.

Seeking social contact might as well be a plausible explanation for both pigs’ and dogs’ proximity seeking behaviour towards their caregiver. The idea behind the short social isolation before the test was to enhance similar motivation to social contact in both pigs and dogs. The increased preference of both species to stay in proximity of their caregiver rather than the familiar object, together with the earlier approach to the caregiver compared with the object, points to a higher incentive value of the social stimulus over the possibility of a food reward, as it has been also described in young wolves and dogs^14,49^. This is of special interest in pigs, as they are naturally highly motivated for food, spending almost one third of their daily routine foraging in nature^50,51^. However, we cannot rule out the possibility that the preference for the caregiver over the familiar object was driven by food motivation rather than by social reinforcement, as normally the owners feed the animal. Since there was no food in the food bowl, it might have been more advantageous to stay in proximity of the caregiver. Interestingly, neither the pigs nor the dogs showed preference for their caregiver when he/she was paired with a stranger and they did not approach the caregiver earlier than the stranger. Furthermore, both pigs and dogs spent more time in total and in physical contact with the stranger than with the familiar object. It can be argued that the unfamiliar social stimulus might have evoked higher exploratory behaviour in both species than the presence of a non-social familiar stimulus.

However, although both family pigs and dogs sought for the proximity of their caregiver, we found several differences between their performance. First of all, although the presence of a stranger might have elicited similar exploratory behaviour in dogs and pigs, their behaviour—as reflected in the time spent near the humans—was different in the Caregiver–Stranger condition. Our results revealed the emergence of a preference for overall human proximity in dogs but not in pigs, which might be a species-specific predisposition. This is in line with previous research in which dogs seem to be predisposed to seek human contact and to display a general human preference from 5 weeks of age^14^ that is not restricted to their caregiver. Due to differences in the two species’ domestication history, i.e. dogs—but not pigs—having been intensively selected for their sociability to humans and for cooperative purposes^52^, humans might be a more naturally salient stimulus for family dogs than for family pigs. Until very recently, domestic pigs have been mostly selected for their role as meat stock, and not for their sociability towards humans. This might be also related to how pigs and dogs differently generalize (their previous interspecific social experience) to other humans. As it has been argued before, domestication might have changed dogs’ ability to recognize individuals, making it more flexible, so that they can easily generalize their early experience with familiar humans to unfamiliar ones^14^. This general pattern of dogs’ preference for being close to humans was also reflected in their returning frequency, as they returned more frequently than pigs to the caregiver in both conditions, and both species returned more frequently to the stranger than to the object.

As we have stated above, pigs did not avoid the stranger completely, but in contrast to dogs, they did not show preference for social contact when the stranger was present. In fact, pigs spent more time away from any of the choices during the Caregiver–Stranger condition than during the Caregiver–Object condition. Previous research showed that pigs with previous positive experience with humans tend to show decreased fear responses towards an unfamiliar handler^44^, but they might also face an approach-avoidance conflict when confronted with an unfamiliar human^11^. Family pigs might be in general motivated to explore the stranger, but when confronted with him, the novel social stimulus might evoke some fearfulness^53^, which is manifested in a general social stimulus-avoiding behavioural response. Exploratory behaviour towards the environment is regarded as a typical response in pigs^53^ which normally reflects curiosity and good welfare^54^, but it has also been found to increase in pigs experiencing frustration^37^. Our pig subjects may have been curious to explore the stranger but at the same time also potentially fearful of her, which could have resulted in mild frustration and consequently, in exploratory behaviour away from the stranger. Nevertheless, as we haven’t observed any other frustration related behaviours this statement remains speculative. On the other hand, pigs’ lower return frequencies together with the later approach to any of the targets, compared with dogs, might reflect that pigs are in general slower than dogs^55,56^—although they can be very fast in short bouts (e.g. during play^57^). However, we can speculate that the observed return frequencies of pigs might also reflect that once pigs have made a decision (e.g. staying away from the humans, or staying close to the caregiver), they did not change their choice afterwards. We should keep in mind that the found differences between dogs and pigs, especially in the Caregiver–Stranger condition, might be linked to pigs being preys and dogs being predators. For a prey animal, a new social stimulus might be perceived as a higher potential threat so it might be a better strategy to stay away from it^58^ or to stay close to the caregiver where they might be more secure.

The species-specific socialization period might also be determinant for the generalization process in both species. Even though our subjects were the same age, this does not guarantee the same behavioural state in development. One outcome of domestication is the prolonged sensitive period for socialization in dogs^59^, compared to wolves which might be advantageous for recognizing broader range of stimuli (e.g. humans) as familiar, and consequently showing decreased fear reactions towards them. Family pigs’ socialization period starts at week 2 of age^60^ and ends earlier during development, at around week 10^38,61^, shortening the time window for recognizing new stimuli as familiar. Previous research, under farm conditions, reported early positive handling of pigs before 4 weeks of age, which might be important for the generalization process to unfamiliar handlers. Our subjects, although they have been exposed regularly to strangers, were adopted by their human family not sooner than 8 weeks of age, which might have influenced the present results.

Furthermore, we also observed species differences in other human-oriented behaviours and in vocal behaviour. In the first place, we found that pigs in the Caregiver–Object condition showed a preference for being not only near to the caregiver but also in physical contact with her and they tended to do it more than dogs. During intraspecific interactions, pigs perform a broad range of physical contacts^62,63^, tending even to sleep in close body contact with familiar conspecifics^51^. This species-specific social behaviour might also be present in interspecific interactions which is in line with previous observations in which pigs showed high motivation to physically contact the handler^35,42,57^. When pigs have a positive experience with humans, they readily perform a variety of human-oriented physical interactions, from playful ones (e.g., biting and shaking the human^37^) to calmer ones (e.g., lying in contact with the human^61^). Our findings are in line with the above, not only because family pigs showed a clear tendency to be in contact with their caregiver, but also because although they contacted the stranger they did it to a lesser extent than dogs, supporting the idea that the new stimulus might have been perceived as a potential threat. Furthermore, although it was not our aim to distinguish between the different types of physical interactions that the pigs performed we could identify some. Notably, we observed our pig subjects rooting and nosing the caregiver, which are considered to be for social recognition and affiliative behaviours^62^. Additionally, more than half of the pigs climbed towards the owner's lap which is an expression of a calm state and high affinity for the handler^64^.

Second, all pigs, but not all dogs, vocalized in both conditions, similarly as we have reported in previous studies^35^. Although we found no evidence that the vocalizations had any interspecific communicative function, pigs tended to vocalize more when they were away than close to the social partners. Importantly, although in the present study it was not our aim to perform a qualitative analysis, the majority of vocalizations emitted by the pigs were the so called “grunts”. Pigs are reported to grunt across a wide range of contexts, social and non-social^65^. Grunts are also associated with locomotor activity^66^ and exploratory behaviour^67^. The grunting rate might increase under increased arousal (e.g. fear or frustration)^68^, as well as during isolation^69^. There are two ways to explain our observations. Firstly, one could argue that pigs grunted more while they were away because they were engaged with exploring the novel environment. Alternatively, young piglets, at a large distance from the owner, could find themselves in isolation, thus they might grunt as a contact call. However, these interpretations remain speculative as our quantitative results do not provide further evidence for the interspecific communicative nature of the pigs’ vocalizations.

A few factors may also limit the interpretation of our results. One of the main goals of the present study was to assure similar experience with humans to both species, to rule out the possibility that any of our findings about species difference were due to major differences in socialization background. However, even though we supervised our pig subjects’ daily home routines, we cannot prove that both species have been provided with the same amount of and qualitatively similar human interaction and experiences. Although pigs regularly met new people, we cannot rule out the possibility that family dogs were more familiar with meeting strangers than the pigs, which led to broader generalisation potential in dogs toward humans. In addition, very early experience of the subjects before adoption at 8 weeks, might be also important for the found differences. Furthermore, the fact that the two humans were relatively close to each other during the Caregiver–Stranger condition might allow for alternative explanations. One could also argue that pigs stayed away from both humans instead of being in proximity of their caregiver because they simply avoided being anywhere near the stranger. Along with this, despite our efforts to balance the gender of the stranger with the gender of the caregiver in some cases it was not possible, similarly as in previous studies^70^. This could cause a difference in the behaviour towards the stranger in those cases, however we found no differences in the behaviour of those subjects compared with the rest of the subjects (see Supplementary material). Finally, there are two factors that might limit the generalizability of our findings. The first is the low sample size, which was a consequence of the strict inclusion criteria (but see other pig’s behaviour studies with similar sample sizes^42,43^). The second is the fact that most pigs belonged to the same Minnesota miniature breed (see Supplementary material Table S1 for details). However, individual variability in the observed behaviours was not higher among dogs (that belonged to different breeds) than pigs.

To sum up, we found that after intense socialization juvenile pigs, as family dogs, displayed proximity seeking behaviour towards their caregiver. But species-specific predispositions and development seem to influence the nature of this behaviour and also how both species generalize early interspecific social experience to other humans. To what extent early human socialization (before 8 weeks) could promote an overall preference for humans in young family pigs similar to that showed in family dogs, needs further investigations. Future research should also focus on the different reactions towards the caregiver and unfamiliar humans of adult family pigs, in comparison with family dogs, in different contexts. Along with these, our results are informative with regard to the potential of the miniature pig’s usage in comparative ethological research and they contribute to the field of human–animal relationships as well. As a first study providing insight to the behaviour of companion pigs towards their caregiver and a stranger, the findings presented here may be of applied, welfare-related importance and may prove to be useful when socializing pigs as family animals.

## Supplementary information

Supplementary Information.

Supplementary Dataset.

## Data Availability

All data generated and analysed during this study are included in this published article and its Supplementary Information file (see Supplementary Data).
