# Supplementary material for: Human proximity seeking in family pigs and dogs
Source: Sci Rep. 2020 Nov 30;10:20883. doi: 10.1038/s41598-020-77643-5 (PMC7705753; doi:10.1038/s41598-020-77643-5)
Supplement: Supplementary file 1 — Supplementary Information. [file 41598_2020_77643_MOESM1_ESM.docx]

**Supplementary Information for**

**Human proximity seeking in family pigs and dogs**

**Authors and affiliations**

Paula Pérez Fraga^1,2^ , Linda Gerencsér^1,2*^, Attila Andics^1,2^

^1^ Department of Ethology, Eötvös Loránd University, Budapest, Hungary

^2^ MTA-ELTE ‘Lendület’ Neuroethology of Communication Research Group, Hungarian Academy of Sciences – Eötvös Loránd University, Budapest, Hungary

*Correspondence: [pauliperezfraga@gmail.com](mailto:pauliperezfraga@gmail.com)

ORCID ID: <https://orcid.org/0000-0001-5625-3224>

Tel: +34 652440760

Address: Department of Ethology, Eötvös Loránd University, 1117 Budapest, Pázmány P. s. 1/C, Hungary

**Detailed subject information**

| **Subject ID** | **Species** | **Birth date** | **Test age (months)** | **Gender** | **Breed** |
| --- | --- | --- | --- | --- | --- |
|  |  |  |  |  |  |
| 1 | Pig | 14/08/2017 | 5 | male | Minnesota Mixed |
| 2 | Pig | 19/08/2017 | 5 | male | Minnesota Mixed |
| 3 | Pig | 23/08/2017 | 5 | male | Minnesota |
| 4 | Pig | 23/10/2017 | 3 | male | Minnesota |
| 5 | Pig | 23/10/2017 | 3 | fame | Minnesota |
| 6 | Pig | 23/10/2017 | 3 | female | Minnesota |
| 7 | Pig | 25/04/2017 | 3 | female | Minnesota |
| 8 | Pig | 05/04/2018 | 3 | male | Minnesota |
| 9 | Pig | 25/03/2018 | 4 | male | Minnesota |
| 11 | Dog | 08/03/2018 | 3 | male | Poodle |
| 12 | Dog | 08/03/2018 | 3 | male | Poodle |
| 13 | Dog | 08/03/2018 | 3 | female | Poodle |
| 14 | Dog | 28/03/2018 | 4 | female | Border collie |
| 15 | Dog | 23/05/2018 | 3 | female | Schnauzer |
| 16 | Dog | 25/05/2018 | 3 | female | Belgian malinois |
| 17 | Dog | 24/06/2018 | 4 | male | Komondor |
| 18 | Dog | 25/06/2018 | 3 | female | Border terrier |
| 19 | Dog | 15/06/2017 | 5 | male | Pumi |
| 20 | Dog | 20/09/2018 | 4 | female | Border Collie |
| 21 | Dog | 20/09/2018 | 4 | male | Border Collie |
| 22 | Dog | 12/10/2018 | 4 | female | Rough Collie |

**Table S1.** Subject information

| **Contrast** | **Estimate** | **SE** | **df** | **t ratio** | ***P* value** |
| --- | --- | --- | --- | --- | --- |
| Dog, C-O - Pig, C-O  Dog, C-O - Dog, C-S  Pig, C-O - Pig, C-S  Dog, C-S - Pig, C-S | -1.166  -0.299  1.398  0.531 | 0.623  0.475  0.548 | 34.43  19  19 | -1.872  -0.630  2.511 | 0.259  0.921  0.084 . |
|  |  | 0.623 | 34.43 | 0.854 | 0.828 |

Note. Significance code: '.' < 0.1. Tukey method for *P* value adjustment.

**Table S2.** *P* values and related parameters of the post hoc tests for the contrasts on ‘Contact caregiver’

| **Contrast** | **Estimate** | **SE** | **df** | **t ratio** | ***P* value** |
| --- | --- | --- | --- | --- | --- |
| Dog, C-O - Pig, C-O  Dog, C-O - Dog, C-S  Pig, C-O - Pig, C-S  Dog, C-S - Pig, C-S | -0.313  -0.151  0.232  0.070 | 0.126  0.088  0.101 | 32.04  19  19 | -2.485  -1.716  2.288 | 0.082 .  0.343  0.136 |
|  |  | 0.126 | 32.04 | 0.558 | 0.944 |

Note. Significance code: '.' < 0.1. Tukey method for *P* value adjustment.

**Table S3.** *P* values and related parameters of the post hoc tests for the contrasts on ‘Contact ratio for caregiver’

| **Contrast** | **Estimate** | **SE** | **df** | **t ratio** | ***P* value** |
| --- | --- | --- | --- | --- | --- |
| Dog, C-O - Pig, C-O  Dog, C-O - Dog, C-S  Pig, C-O - Pig, C-S  Dog, C-S - Pig, C-S | -6.0  6.733  -59.333  -72.067 | 34.705  16.127  18.621 | 24.36  19  19 | -0.173  0.418  -3.186 | 0.998  0.975  0.023 ^*^ |
|  |  | 34.705 | 24.36 | -2.077 | 0.189 |

Note. Significance code: '*' < 0.05. Tukey method for *P* value adjustment.

**Table S4.** *P* values and related parameters of the post hoc tests for the contrasts on time spent ‘Away’

| **Fixed effects** | **Estimate** | **Std. Error** | ***z* value** | ***P* value** |
| --- | --- | --- | --- | --- |
| Species  Condition  Species * Condition | -0.816  -0.042  -0.122 | 0.27  0.167  0.327 | -3.026  -0.252  0.373 | 0.003 ^**^  0.801  0.709 |

Note. Significance code: '**' < 0.01.

**Table S5.** *P* values and related parameters for the main effects and their interaction on ‘Return to caregiver’ frequency

| **Fixed effects** | **Estimate** | **Std. Error** | ***z* value** | ***P* value** |
| --- | --- | --- | --- | --- |
| Species  Condition  Species * Condition | -0.841  0.617  -0.712 | 0.407  0.213  0.486 | -2.066  2.898  -1.465 | 0.039 ^*^  0.004 ^**^  0.143 |

Note. Significance codes: '**' < 0.01; '*' < 0.05.

**Table S6.** *P* values and related parameters for the main effects and their interaction on ‘Return to stranger/object’ frequency


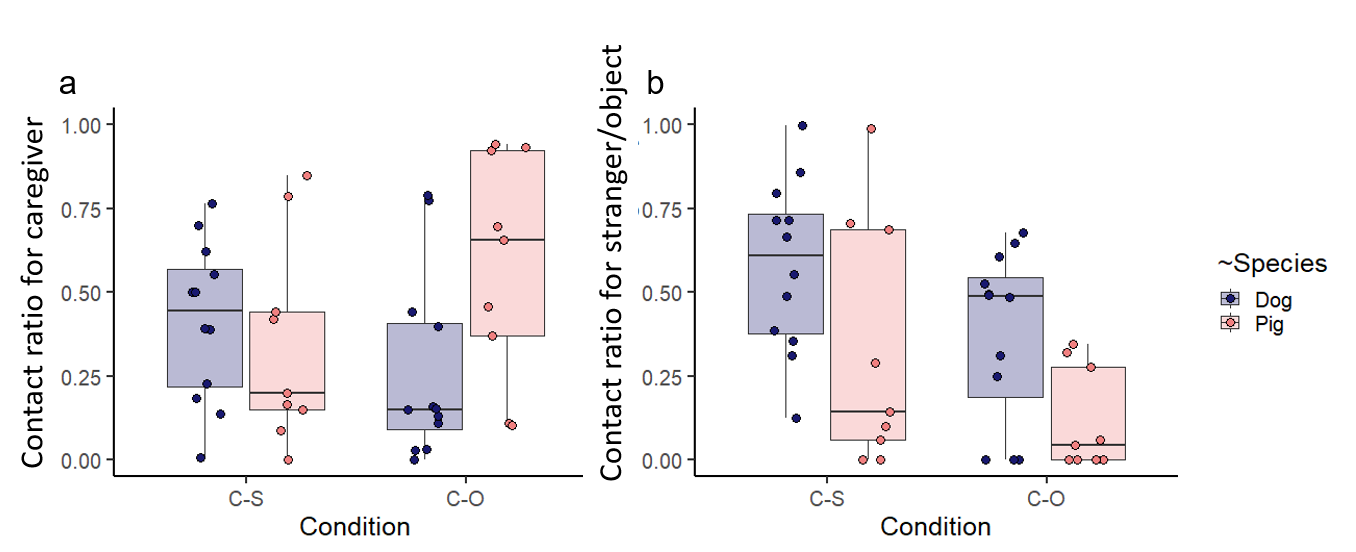


**Figure S1.** Ratio of time spent in physical contact out of the total time spent near (within a distance of 40cm) with a) the caregiver and b) the stranger/object for both species in the two conditions. “C-S” stands for Caregiver-Stranger condition and “C-O” for Caregiver-Object condition. The line across the box represents the sample median, the box represents the interquartile range, and the whiskers show the smallest and largest values (excluding outliers). The dots represent the individual data points.

**Caregiver-Stranger gender match/mismatch**

We found no differences between the behaviour of pigs with caregiver-stranger gender mismatch and pigs with caregiver-stranger gender match; we used Wilcoxon-rank-sum tests to compare their behaviour along the variables time spent ‘Near stranger’ (W = 6, P = 0.52),  time spent ‘Near caregiver’ (W = 9, P = 1) and time spent ‘Away’ from any of the choices (W = 15, P = 0.17).
